# Trapping and manipulation of nanoparticles using multifocal optical vortex metalens

**DOI:** 10.1038/s41598-017-14449-y

**Published:** 2017-11-03

**Authors:** Yanbao Ma, Guanghao Rui, Bing Gu, Yiping Cui

**Affiliations:** Advanced Photonics Center, Southeast University, Nanjing, 210096 China

## Abstract

Optical trapping and manipulation have emerged as a powerful tool in the biological and physical sciences. In this work, we present a miniature optical tweezers device based on multifocal optical vortex metalens (MOVM). The MOVM is capable of generating multiple focal fields with specific orbital angular momentum at arbitrary position. The optical force of the vortex field exerted on both high-refractive-index particle and low-refractive-index particle are analyzed. The simulation results show that the two kinds of dielectric particles can be trapped simultaneously. Besides, it is also feasible to manipulate plasmonic nanoparticles even under the resonant condition, which is realized by constructing a 4Pi focusing system with metalenses. Moreover, the metalens can be made into an array format that is suitable for trapping and manipulating various nanoparticles with diverse motion behaviors. The work illustrates the potential of such optical tweezers for further development in lab-on-a-chip devices, and may open up new avenues for optical manipulation and their applications in extensive scientific fields.

## Introduction

Since Ashkin and his colleagues reported the first stable three-dimensional optical trapping^1^, which is created using radiation pressure from a single focused laser beam, optical tweezers has become an important tool for research in the fields of biology and physical chemistry^2^. Generally, Gaussian beams with spatially homogeneous state of polarization are used to trap dielectric particles with refractive index higher than surrounding medium. In addition, highly focused optical vortex, azimuthally polarized beam and Laguerre-Gaussian beam are proposed to trap low-refractive-index particle^3–5^. Usually it is difficult to trap both kinds of particles, which requires more complex manipulations of the generated optical vortex beam. Due to strong scattering force and severe optical heating effect, trapping plasmonic particles do not always success, especially when the trapping wavelength is close to the resonance of the particle. Thanks to the advanced optical engineering, generation of optical fields with inhomogeneous spatial distribution in terms of phase, amplitude and polarization become possible, which are helpful to improve the performance of optical tweezers and realize novel optical micromanipulation techniques^6–9^. For example, negative scattering force has been reported for some Bessel beams^10,11^, enabling to trap plasmonic nanoparticle even under the resonant condition by tailoring the spatial distribution of the vectorial optical illumination^12,13^.

With rapid development of the electronics industry, miniature optical devices are highly desirable. Traditional optical tweezers involves the use of bulky optical elements and complicated procedures, making it difficult to be integrated into a compact platform. In recent years, metasurface is considered as a promising 2-dimensional metamaterial to design integrated optical devices by controlling electromagnetic wave’s phase, amplitude, and polarization state in a desired manner^14–18^. Varieties of metasurface devices have been fabricated to demonstrate their powerful ability. For example, plasmonic tweezers based on the excitation of surface plasmons on a plasmonic device exhibits enhanced attractive force for various kinds of nanoparticles^19–21^. However, due to the evanescent nature of the surface plasmons, it is even inevitable that the particle would has physical contact with the metallic surface, increasing the risk of sample contamination. Besides, due to the strong near-field strength of the surface plasmons wave, the stability of the optical trapping would be dramatically decreased by the heating effect, which may also bring thermal damage to the samples. Recent progresses on far-field plasmonic trapping have been demonstrated to overcome these drawbacks^22^, however, the available trapping force is only within the range of sub-piconewton to 1pN, restricting its applications in assembling or more complex cellular processes of macromolecules, cell adhesion and contraction that requires much larger forces^23,24^. In this paper, we proposed a novel miniature optical tweezers that is constructed around a multifocal optical vortex metalens (MOVM). The MOVM consists of several sub-lenses, which is capable of focusing circularly polarized light into multiple vortex fields with opposite spin at arbitrary plane. The orbital angular momentum of each focal field can be controlled by the geometrical topological charge of the metalens, enabling not only the stable trap of both high-refractive-index and low-refractive-index particles simultaneously, but also the manipulation of nanoparticles by refractive index. Additionally, a 4Pi optical tweezers is designed with two cascading metalenses, which is capable of trapping plasmonic nanoparticle even under the most challenging conditions. The considerably enhanced optical force is helpful to relieve the thermal effect, overcoming the ultimate obstacle that prevents stable trapping of plasmonic nanoparticle near the resonant wavelength.

## Results

### Design of the MOVM

**Figure 1:**
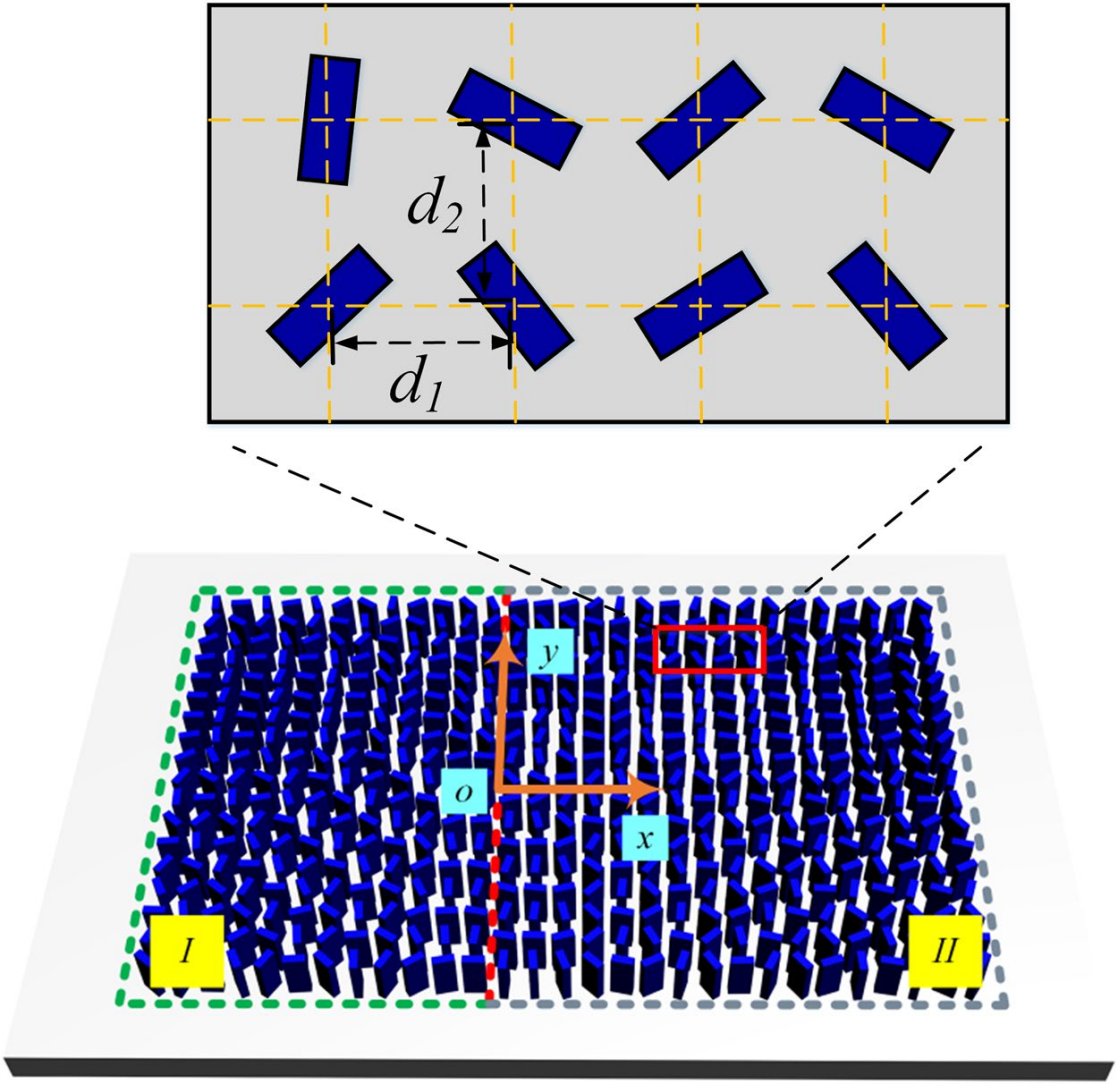
Schematic of the bifocal optical vortex metalens.

Figure 1 shows the schematic of a transmissive planar MOVM. A set of TiO_2_ nanofins fabricated on a glass substrate are employed as building blocks. Due to the structural birefringence, the effective index of the naonfin would be different for two orthogonal polarizations normal to the propagation direction^25^. By choosing the nanofin with length, width and thickness of 250 nm, 95 nm and 600 nm, respectively, its functionality is designed to be a half-wave plate working at 532 nm. It has been demonstrated that the incident circular polarization (CP) can be converted into cross-CP light with conversion efficiency as high as 90%, obtaining the abrupt phase change required to tailor the optical wave-front. Consequently, the nanofin is capable of significantly decreasing the background interference and increasing the purity of desired cross-CP light.

In order to achieve the desired focusing properties of the MOVM, two distinct phase profiles including a spherical convex lens and a vortex lens are incorporated for this ultrathin flat lens:

1 $${\phi }_{s}(x,y)=\frac{2\pi }{{\lambda }_{d}}(f-\sqrt{{x}^{2}+{y}^{2}+{f}^{2}}),$$

2 $${\phi }_{v}(x,y)=m\cdot \arctan (\frac{y}{x}),$$

where *λ*_*d*_ is the designed wavelength, *x* and *y* are the coordinates of each nanofin, ƒ is the focal length, and *m* is the geometrical topological charge of the metalens. Therefore, the secondary wave emerging from the metalens can constructively interfere at the focal plane to produce the focused optical vortex with specific topological charge.

It is crucial that the phase change for cross-CP light can be altered smoothly from 0 to 2*π*. The individual orientation angle of a given nanofin, with respect to the *x*-axis, controls the abrupt phase change of the cross-CP scattered light. The required phase profiles are achieved by rotating each nanofin, which would introduce a geometric phase equal to twice the rotation angle *θ*_*t*_. Consequently, the required phase map given in Eqs (1) and (2) can be realized for normal incidence by rotating each nanofin at a given coordinate (*x*, *y*) by an angle

3 $${\theta }_{t}=\frac{\pi }{{\lambda }_{d}}(f-\sqrt{{x}^{2}+{y}^{2}+{f}^{2}})+\frac{m}{2}\cdot \arctan (\frac{y}{x}).$$

In this way, the required phase map is realized for normal incidence. To create optical vortex metalens with multiple focus, nanofins are arranged in rectangular with lateral (*d*_1_) and vertical (*d*_2_) varying orientations (shown in the inset of Fig. 1) in two sub-lenses (marked by I and II in Fig. 1). Each sub-lens is able to focus incident collimated Gaussian beam into a vortex beam with well-defined topological charge at specific focal plane. As an example, a bifocal optical vortex metalens with focal length of 2 *µ*m is designed. The lateral and vertical distances between the two neighboring elements are both optimized to be 325 nm. The geometrical topological charge and the radius are set to be (0, 3.4 *µ*m) and (2, 9.9 *µ*m) for sub-lens I and II, respectively. The numerical aperture (NA) is defined as NA = *n*_*s*_·sin[tan^−1^(*D*/2 *f*)], where *D* is the diameter of the sub-lens, and *n*_*s*_ is the refractive index of the surrounding medium in the focal region (water, *n*_*s*_ = 1.33). Consequently, the NAs of the sub-lens I and II are 1.14 and 1.3, respectively. Note that different radii of the sub-lenses are chosen in this case purposely in order to achieve focal spots with nearly the same peak intensity. The NA of the metalens can be further increased by introducing more 2*π* periods, giving rise to better focusing properties such as smaller focal spot sizes and stronger focal fields. It is worthy of noting that the orbital angular momentum of the focal field arises from the geometrical topological charge of the metalens, rather than the incident light itself. Since the incident light has uniform phase distribution, there is no requirement on the alignment between the metalens and the illumination, enabling the generation of multiple focal spots by dividing the metalens into several sub-lenses.

**Figure 2:**
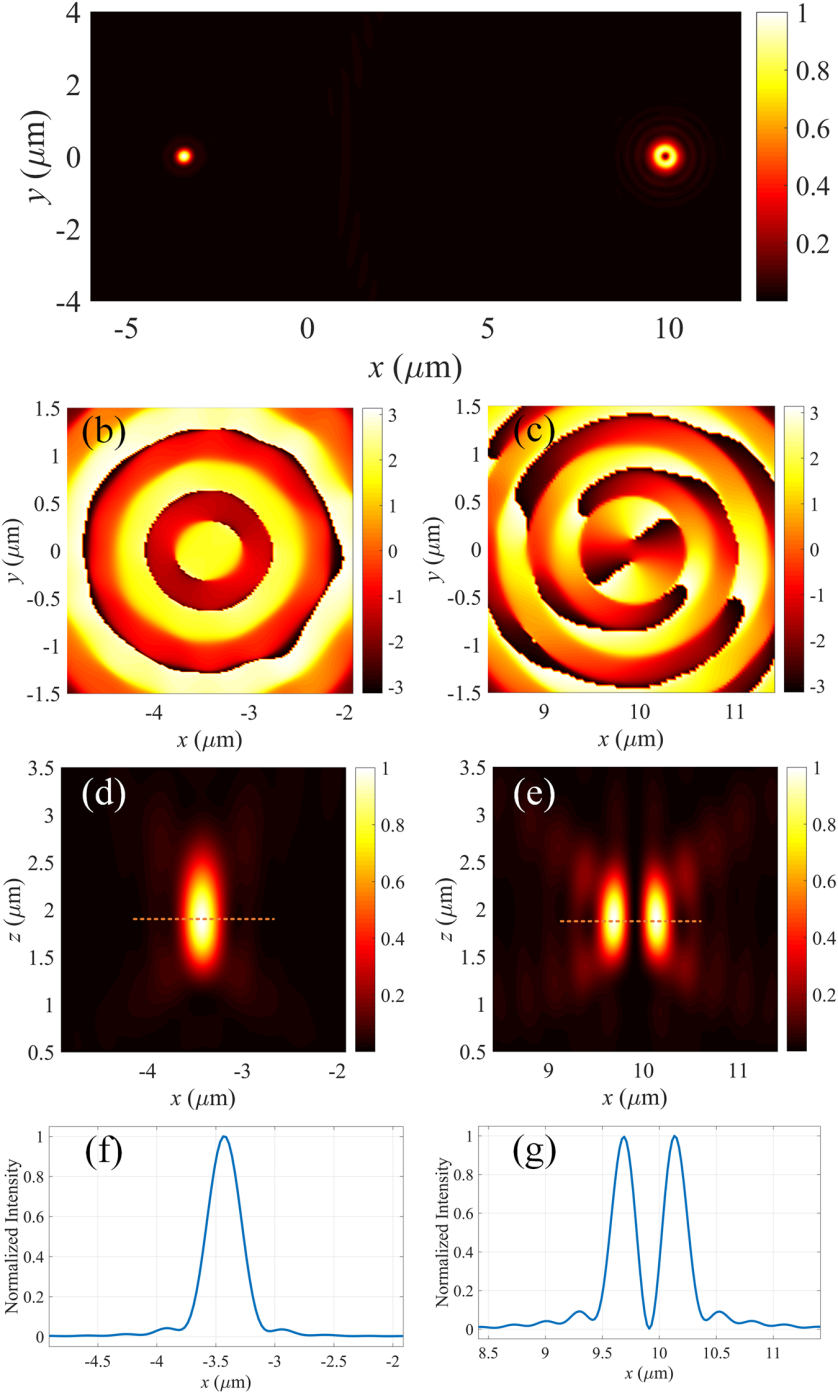
Intensity distribution of the focal field at *λ* = 532 nm (**a**) in the *x*-*y* plane (*z* = 2 µm) and (**d**,**e**) in the *x*-*z* plane (*y* = 0). *E*_*x*_ phase distribution of the bifocal metalens with geometrical topological charge of (**b**) 0 and (**c**) 2, respectively. (**f**,**g**) The line-scan of the distribution in the focal plane for (**d**,**e**).

In order to study the characteristics of the MOVM, a three-dimensional model has been built with finite-difference time-domain (FDTD) method. By illuminating the MOVM with right-hand circularly polarized light, the intensity profile of the focal field in the transverse plane (*z* = 2 *µ*m) is shown in Fig. 2(a). As expected, two focal spots including one solid shape and one doughnut shape with dark center are obtained. The topological charges of the focal fields are 0 and 2, respectively, which can be easily found out by the corresponding phase distributions shown in Fig. 2(b,c) and (d)–(g) show the focal field patterns of the MOVM in the longitudinal plane (*y* = 0) and the corresponding line-scans along the *x*-axis. It can be seen that the focal length of the metalens is about 2 *µ*m, which is consistence with the initial design. The Full-Width-Half-Maximum (FWHM) of the focal fields are found to be about 320 nm and 280 nm for the central peak and the dark center, respectively, revealing the high NA and the great focusing performance of the proposed MOVM. Note that not only the topological charge but also the position of each focal field can be controlled by the phase profile of the metalens. In this case, the two sub-lenses are capable of producing focal fields propagating normal to the substrate. Consequently, the lateral distance between the foci should be the distance between the centers of the sub-lenses (13.325 *μ*m).

### Trapping and manipulating nanoparticles using MOVM

**Figure 3:**
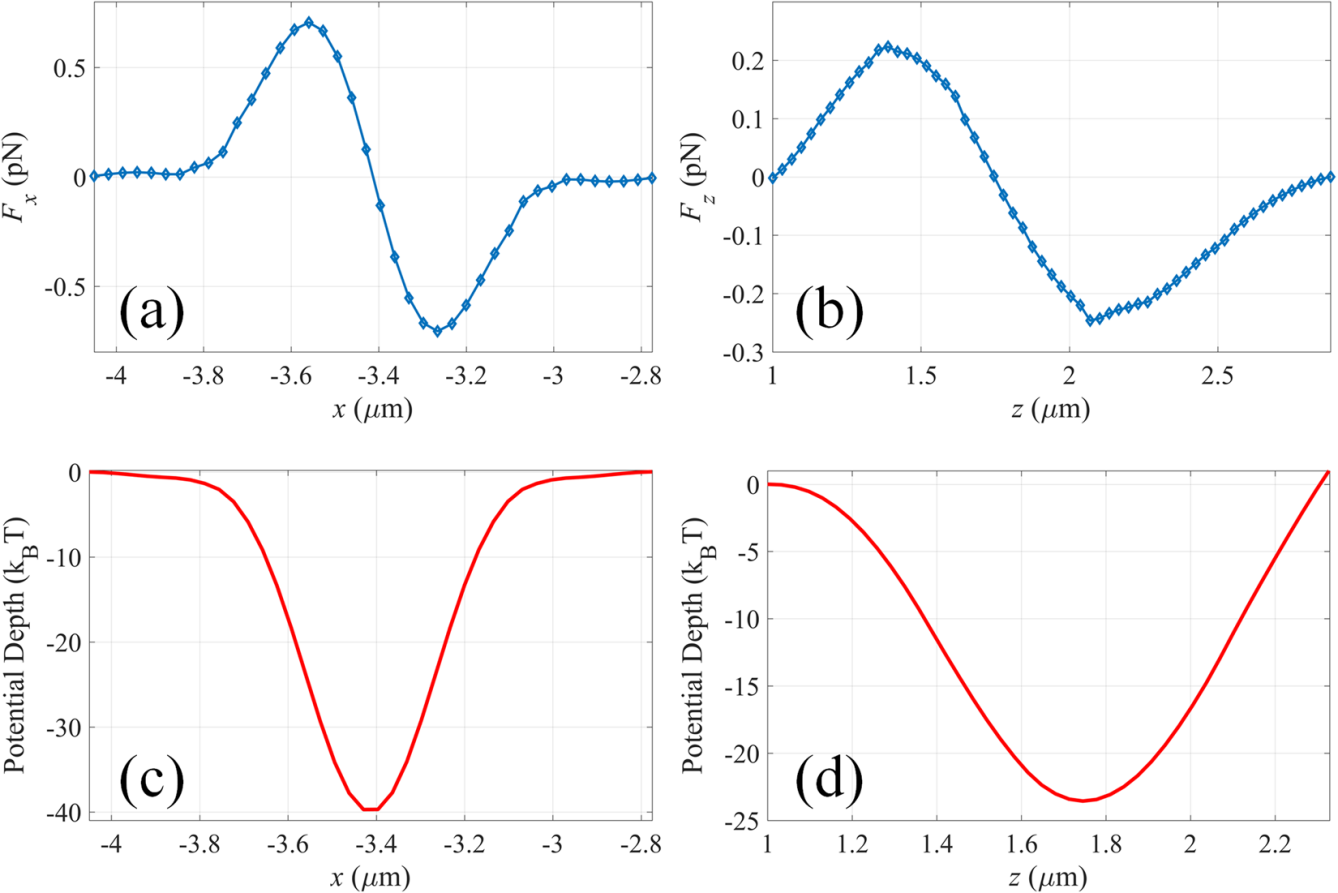
Optical force exerted on a 100 nm (radius) high-refractive-index dielectric nanoparticle along (**a**) *x*- and (**b**) *z*-axis of the focal field with topological charge of 0. Distribution of the corresponding potential depth along (**c**) *x*- and (**d**) *z*-axis.

Due to the incoming optical flux, the movement of a nanoparticle within the focal region of the MOVM would depend on the induced optical force. By integrating the Maxwell stress tensor (MST) over the particle surface, the time-average force <F> (including both gradient force and scattering force) exerted on the particle can be expressed as^26^:

4 $$\langle F\rangle =\int \{\frac{\varepsilon }{2}\mathrm{Re}[(E\cdot n){E}^{\ast }]-\frac{\varepsilon }{4}(E\cdot {E}^{\ast })n+\frac{\mu }{2}\mathrm{Re}[\mu (H\cdot n){H}^{\ast }]-\frac{\mu }{4}(H\cdot {H}^{\ast })n\}ds,$$

where *ε* and *μ* are the relative permittivity and relative permeability of the medium around the particle, and *n* is the unit normal perpendicular to the integral area *ds*. Note that both the electric and the magnetic field components required in the MST method are obtained from the FDTD simulation data directly. It is known that solid focal spot is suitable for trapping nanoparticles with refractive index larger than surrounding medium. Considering a dielectric high-refractive-index nanoparticle with radius of 100 nm and refractive index of 1.59 placed around the center of the focal field of sub-lens I, the distributions of the transverse and longitudinal forces exerted on the particle are shown in Fig. 3(a),(b). With the assumption that the input power is 50 mW, the maximal optical forces are found to be nearly 0.8 pN and 0.2 pN along *x*- and *z*-axis, respectively. Clearly, there is an equilibrium point at (*x*, *y*, *z*) = (−3.4 *μ*m, 0, 1.7 *μ*m), corresponding to the position of the peak intensity of the focal field. To evaluate the stability of the optical tweezers, potential depth is estimated by integrating the optical force along the trapping direction $U=-\int F\cdot ds$. Traditionally an optical trap with potential depth larger than *k*_*B*_*T* can be considered as stable, where the temperature *T* is taken to be 293 K. The distributions of the potential depth along the transverse and longitudinal directions are calculated and presented in Fig. 3(c),(d). The potential depth at the equilibrium point are found to be 40 × *k*_*B*_*T* and 23 × *k*_*B*_*T*, respectively along *x*- and *z*-axis, demonstrating the stable trapping of high-refractive-index nanoparticle by the focal field with topological charge of 0.

**Figure 4:**
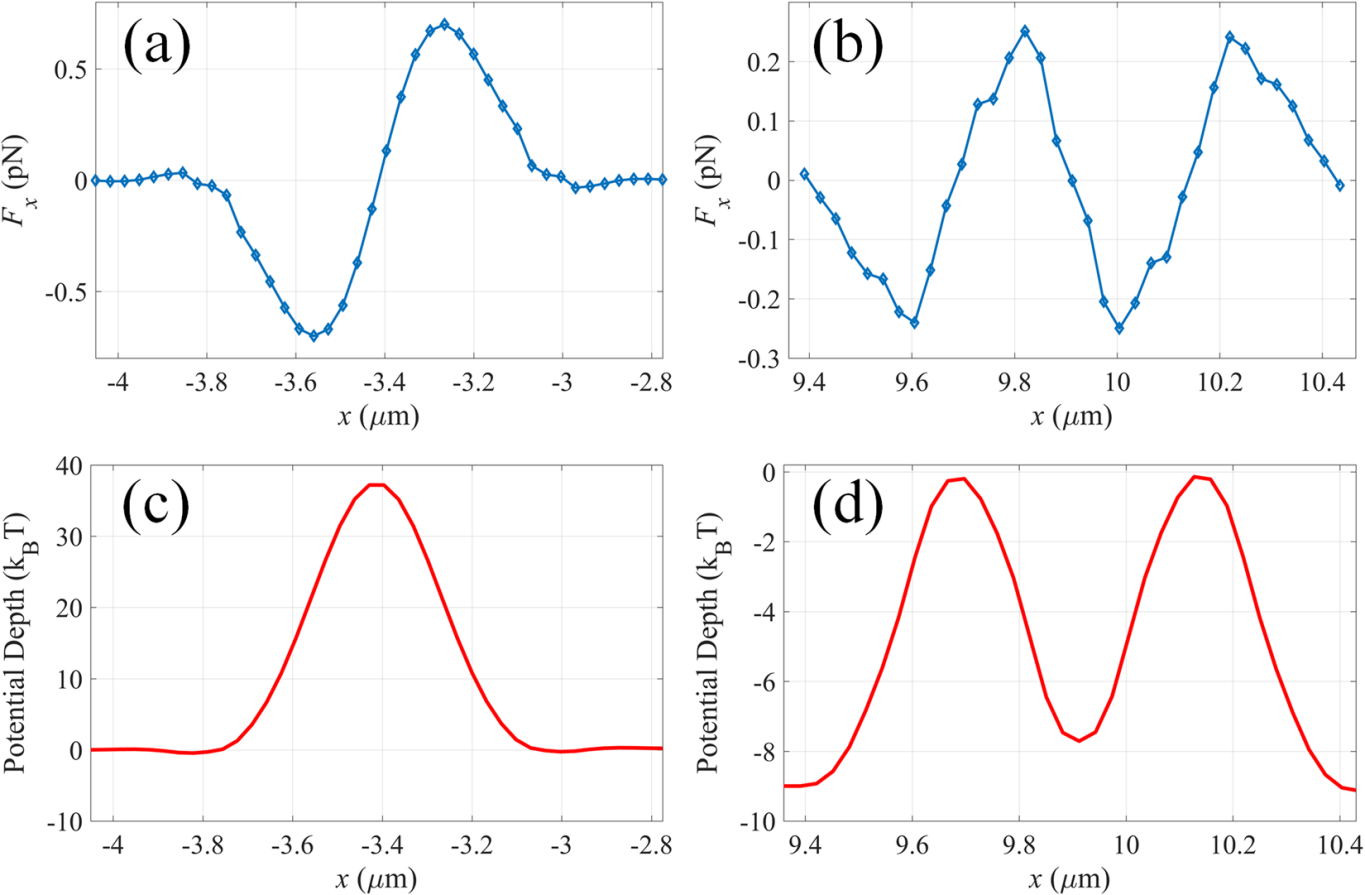
Optical force exerted on a 100 nm (radius) low-refractive-index dielectric nanoparticle along *x*-axis the focal field with topological charge of (**a**) 0 and (**b**) 2. Distribution of the corresponding potential depth of a low-refractive-index nanoparticle along *x*-axis for focal field with topological charge of (**c**) 0 and (**d**) 2.

**Figure 5:**
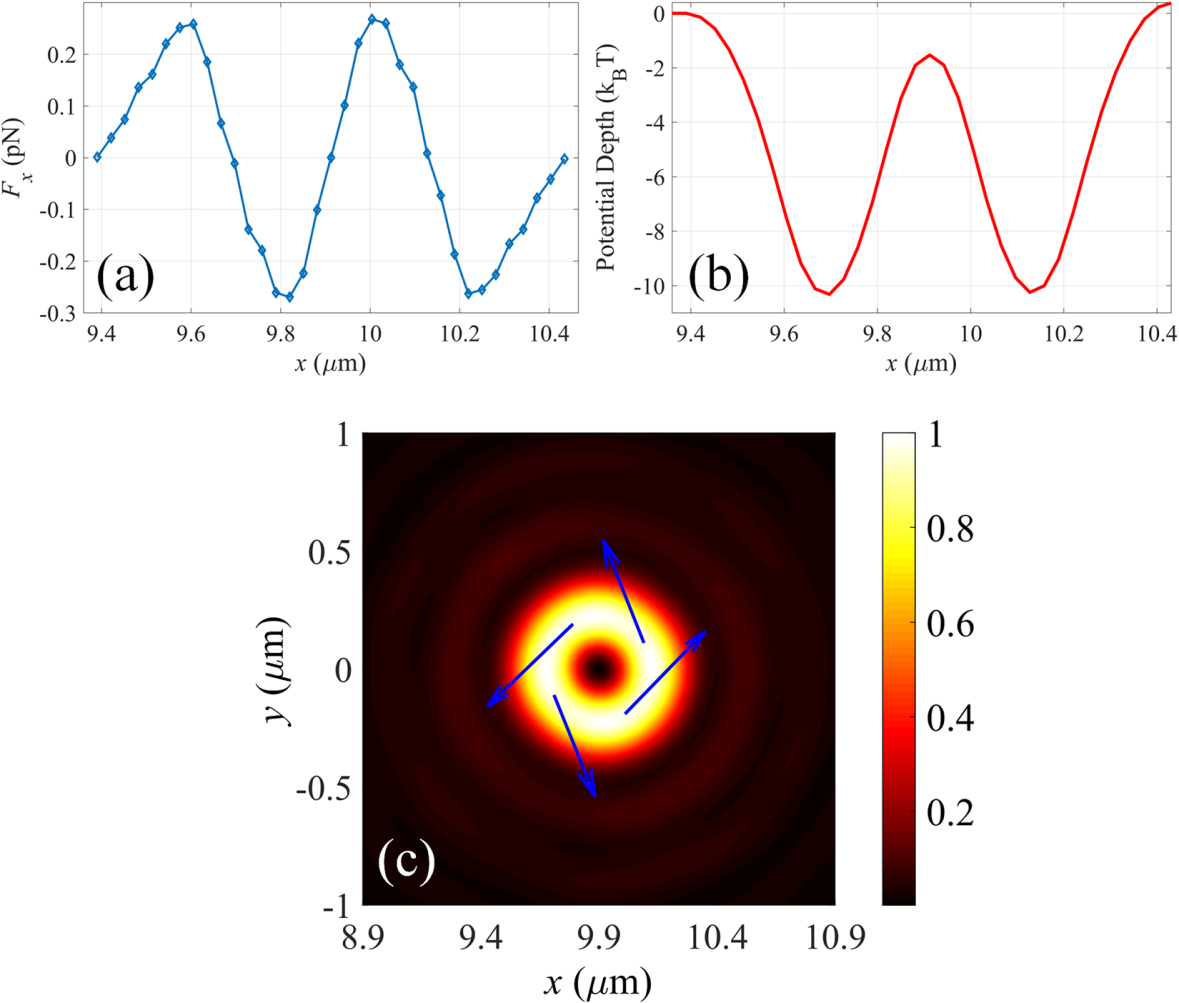
(**a**) Optical force exerted on a 100 nm (radius) high-refractive-index dielectric nanoparticle along *x*-axis the focal field with topological charge of 2. (**b**) Distribution of the corresponding potential depth along *x*-axis. (**c**) Force map superimposed on the intensity distribution of the vector focal field, indicating the anticlockwise rotation of the trapped particle.

For nanoparticle with refractive index (*n* = 1) lower than the surrounding medium, solid focal field would strongly push it away from the focal spot (the corresponding force and potential depth distributions are shown in Fig. 4(a),(c)). In order to trap low-refractive-index particles, doughnut shaped focal spot generated by sub-lens II is adopted. As shown in Fig. 4(b),(d), the particle would be stably confined in the dark center of the vortex field as long as the size of the particle is smaller than that of the dark center of the focal field. It is worthy of noting that the size of the trappable nanoparticles can be increased by increasing the geometrical topological charge of the metalens. Besides, high-refractive-index nanoparticle can also be confined within the annular ring of the optical vortex field (shown in Fig. 5(a),(b)). It is known that tightly focused azimuthally polarized light can only confine the nanoparticle radially but move erratically in the azimuthal direction (Brownian motion)^27^. However, the optical vortex field would force the nanoparticle to circle around the dark center. This phenomenon can be understood by the conservation law of angular momentum in this closed physical system. The orbital angular momentum of the illumination will be inherited by the trapped particle, leading to the rotation of the particle around the center of the light field. To better illustrate it, the force directions at four radial equilibrium positons are indicated by the arrows shown in Fig. 5(c). It can be clearly seen that the particle would anticlockwise rotate with radius of 220 nm.

**Figure 6:**
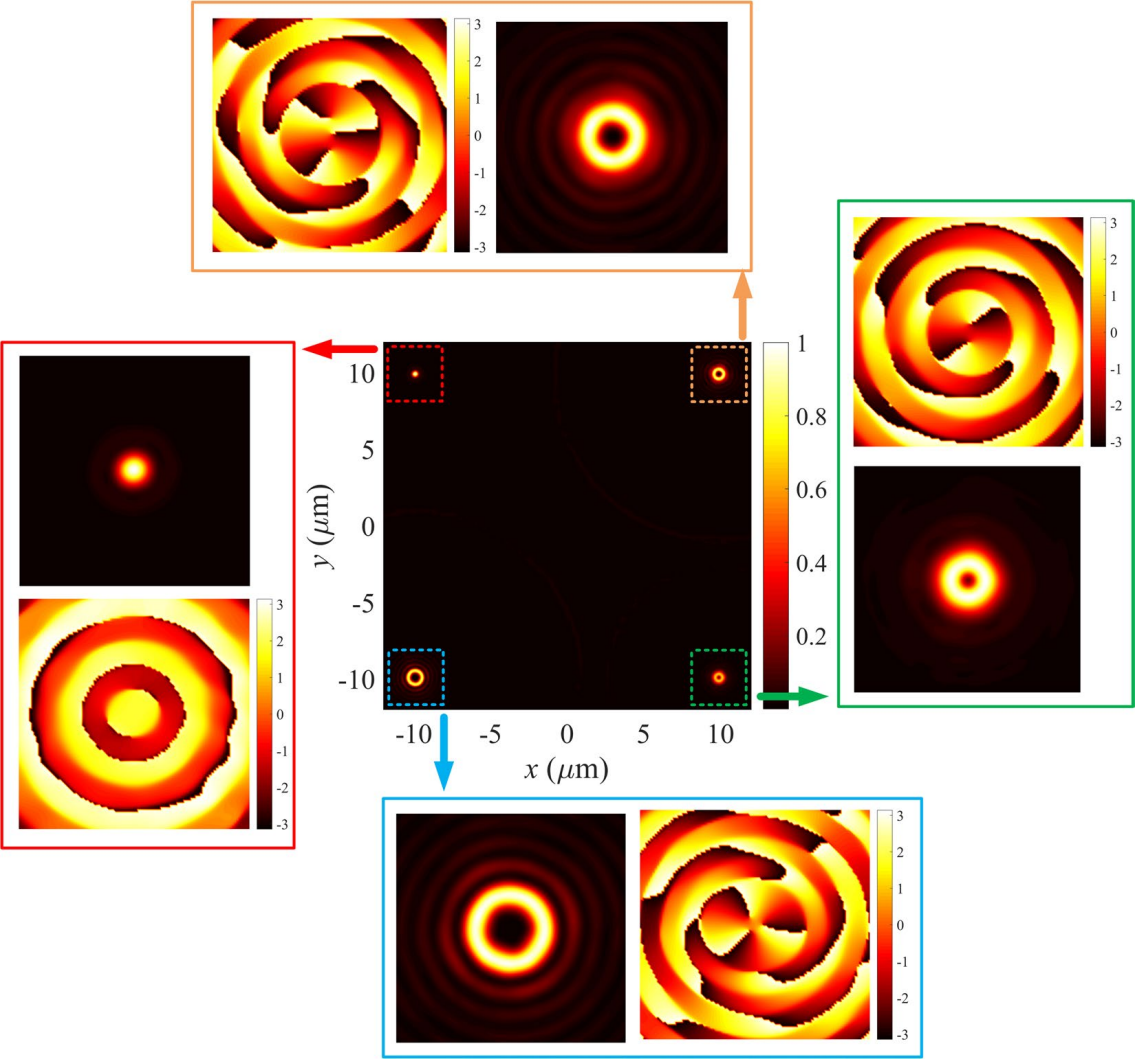
Intensity profile of the MOVM with four sub-lenses. The numerical apertures and geometrical topological charges of the sub-lenses are (0.86, 0), (0.92, 2), (0.98, 3) and (0.98, −3), respectively. The insets show the zoom-in of the intensity and *E*_*x*_ phase distributions of each focal field.

The proposed bifocal optical vortex metalens offers a strategy to trap both kinds of nanoparticles simultaneously. In fact, one key advantage of the MOVM is the elimination of the alignment requirement of the illumination and metalens, enabling its application in optical tweezers with an array format. Figure 6 shows the focal field intensity pattern of a 2 × 2 array that contains different geometrical topological charges. In this case, four focal fields with topological charges of 0, 2 and ±3 are generated, making it feasible to manipulate various kinds of nanoparticles with diverse motion behaviors. It is worthy of noting that the rotation radius and the rotation direction can be adjusted by the absolute value and the sign of the geometrical topological charge, respectively.

### 4Pi focusing system with metalens

**Figure 7:**
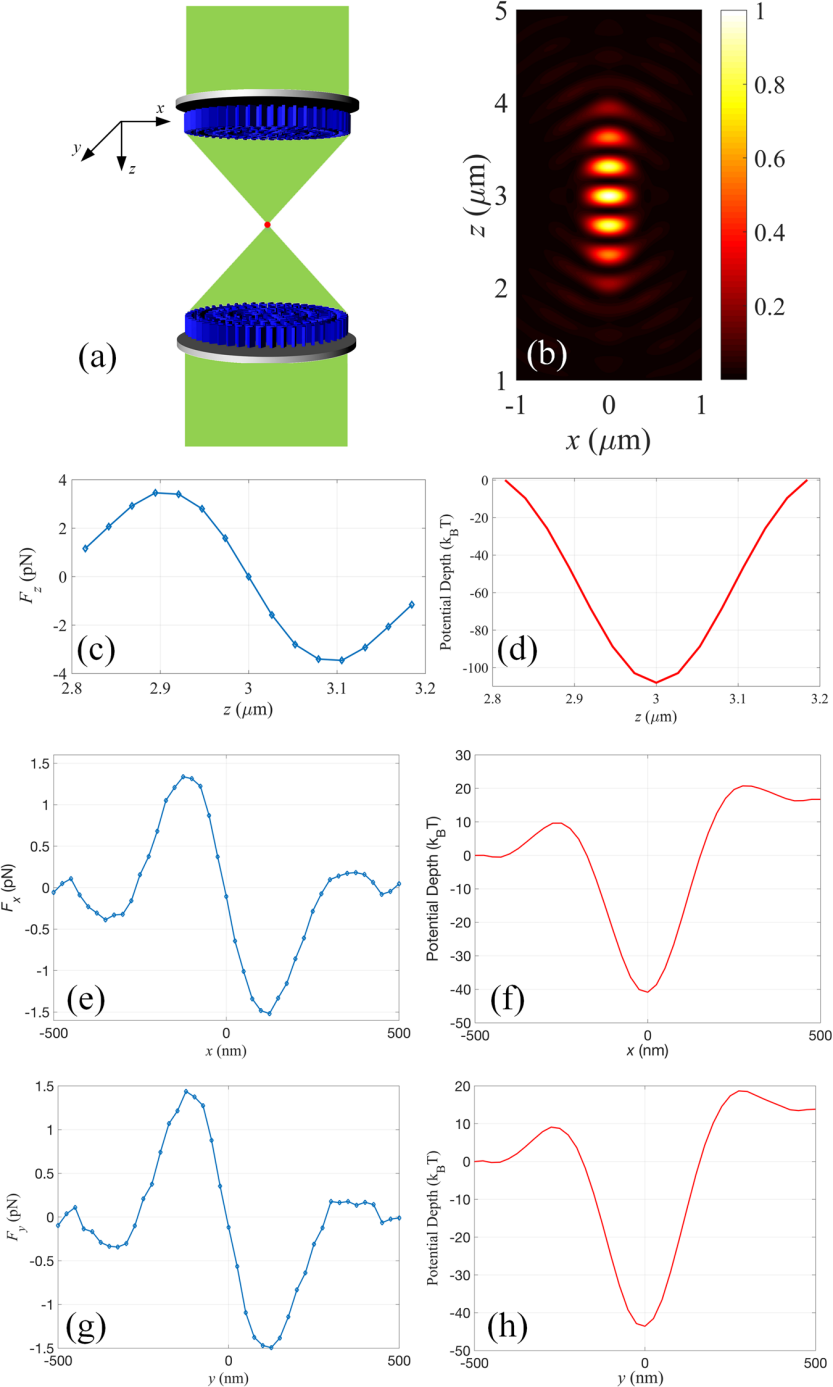
(**a**) Schematic of the optical tweezers consists of two identical metalens with geometrical topological charge of 0. (**b**) Numerical simulation of the intensity distribution in the focal region of the optical tweezers. Optical force exerted on a 50 nm (radius) resonant gold nanoparticle along (**c**) *z*-axis, (**e**) *x*-axis and (**g**) *y*-axis of the optical tweezers. Distribution of the corresponding potential depth along (**d**) *z*-axis, (**f**) *x*-axis and (**h**) *y*-axis.

Different from dielectric ones, trapping plasmonic nanoparticles generally are considered difficult mostly due to the dominating scattering force. In order to increase the trapping efficiency of plasmonic nanoparticle, several methods have been proposed to decrease the magnitude of the scattering force or even reverse the direction of the scattering force to be against the power flow^12,13^. However, these methods involve the use of optical manipulation techniques to generate the required optical complex field, which are difficult to apply in practice. Recently, an optical trapping strategy using 4Pi confocal microscope is proposed to eliminate the axial scattering force^28^. Similarly, it is feasible to construct an optical tweezers by cascading two metalenses, avoiding the use of bulky objective required in a traditional 4Pi focusing system. As shown in Fig. 7(a), two metalenses with the same NA and focal length (3 *µ*m) are placed face-to-face with axial separation of 6 *µ*m. Two light fields with circular polarization normally illuminate the focusing system. In this case, monofocal water-immersed metalens with geometrical topological charge of zero and NA of 0.98 is adopted. Figure 7(b) shows the standing-wave pattern formed by the counter-propagating focusing fields. The focusing fields from the metalenses constructively interfere, leading to further enhanced optical force. To better reveal the performance of the proposed optical tweezers, plasmonic nanoparticle under the resonant condition is considered, corresponding to the most challenging situation to achieve a stable trap. Assuming a water-immersed gold nanoparticle with radius of 50 nm placed near the central focal spot, the trapping wavelength is chosen to be 532 nm, which is close to its both absorption and plasmonic resonances. The input power is set to be 50 mW for each illumination. Figure 7(c),(d) shows the induced optical forces and potential depths along the optical axis. Since the axial scattering force induced by the two focal fields would cancel out each other, the behavior of the resonant gold nanoparticle is primarily determined by the gradient force. Besides, optical forces and potential depths along *x*- and *y*-axis are presented in Fig. 7(e)–(h). Consequently, a stable trap can be formed in three-dimensional space from the force balance point of view. Note that the strength of the interference field would be maximized when the lens-to-lens distance is the sum of their focal lengths, giving rise to the best condition for trapping nanoparticles. Besides, the high focusing efficiency of the optical tweezers enables to sustain the force stability with relatively low input power, which is helpful to relieve the thermal effect that may destabilize the optical trapping. By building an optic-thermal coupling model, the temperature around the resonant gold nanoparticle is simulated to be only about 420 K, which is below the critical temperature for bubble formation (647 K)^29^. Consequently, optical overheating effect can be avoided while maintaining deep enough trapping potential, enabling stable trap of metallic nanoparticle even under the resonant wavelength.

## Conclusions

In summary, we propose an optical trapping and manipulation strategy based on MOVM, which is capable of generating multiple vortex focal fields with specific orbital angular momentum at arbitrary plane. The interaction between the focal field and the nanoparticles is investigated with FDTD algorithm, and the induced mechanical effect is studied using MST method. In order to trap both high-refractive-index and low-refractive-index dielectric nanoparticles simultaneously, a bifocal optical vortex metalens was designed to focus the incident circular polarization into two focal fields with solid and doughnut shape, respectively. Besides, the axial scattering force can be eliminated by a 4Pi focusing system that consists of two cascading metalenses, enabling the stable trap of plasmonic nanoparticles even under the resonant condition. Moreover, no stringent alignment between the illumination and the MOVM is required as long as the illumination is uniform. Such metalens can be made into a two-dimensional array and the orbital angular momentum of each focal field can be controlled by the geometrical topological charge of the metalens, making this MOVM very attractive for trapping and manipulating various kinds of nanoparticles with diverse motion behaviors. It is worthy of noting that fabrication of the proposed metalens is within the capability of the modern nanofabrication techniques^29–32^. This versatile trapping method may open up new avenues for optical manipulation and their applications in various scientific fields.

## Methods

### Simulation method

The full-wave simulations of the characteristics of the devices shown in Figs 1 and 7(a) were performed using Lumerical FDTD solutions. The optical forces were calculated by the *Optical force MST* toolbox available in Lumerical FDTD solutions.
