# Publisher Correction: Trapping and manipulation of nanoparticles using multifocal optical vortex metalens

**DOI:** 10.1038/s41598-018-19647-w

**Published:** 2018-01-15

**Authors:** Yanbao Ma, Guanghao Rui, Bing Gu, Yiping Cui

**Affiliations:** Advanced Photonics Center, Southeast University, Nanjing, 210096 China

Correction to: *Scientific Reports* 10.1038/s41598-017-14449-y, published online 03 November 2017

The original HTML version of this Article contained a typographical error in the publication date ‘03 November 2017’ which was incorrectly given as ‘06 November 2017’. This has now been corrected in the HTML version of the Article.

